# Does Ramadan Intermittent Fasting Affect the Fasting Blood Glucose Level among Type II Diabetic Patients?

**DOI:** 10.3390/jcm12206604

**Published:** 2023-10-18

**Authors:** Yazun Jarrar, Ghasaq Abdul-Wahab, Rami Mosleh, Sara Abudahab, Qais Jarrar, Anas Hamdan, Shurouq Ghalib Qadous, Ruba Balasmeh, Abdulqader Fadhil Abed, Yasmeen Ibrahim, Amin A. Al-Doaiss, Mohammed Ali AlShehri

**Affiliations:** 1 Department of Basic Medical Sciences, Faculty of Medicine, Al-Balqa Applied University, Al-Salt 19117, Jordan; 2 Department of Oral Surgery and Periodontology, College of Dentistry, Al-Mustansiryia University, Baghdad 10052, Iraq; ghasaq.a.abdulwahhab@uomustansiriyah.edu.iq; 3 Department of Pharmacy, Faculty of Medicine and Health Sciences, An-Najah National University, Nablus 00970, Palestine; r.musleh@najah.edu; 4 Department of Pharmacotherapy and Outcomes Science, School of Pharmacy, Virginia Commonwealth University, Richmond, VA 23284, USA; abudahabs@vcu.edu; 5 Department of Pharmaceutical Science, Al-Isra’a University, Amman 11622, Jordan; jarrarq@yahoo.com; 6 Department of Anesthesia and Resuscitation Technology, Faculty of Medicine and Health Sciences, An-Najah National University, Nablus 00970, Palestine; a.hamdan@najah.edu; 7 Department of Nursing and Midwifery, Faculty of Medicine and Health Sciences, An-Najah National University, Nablus 00970, Palestine; sh_qadose@najah.edu; 8 Department of Pharmacy, Faculty of Pharmacy, Al-Zaytoonah University of Jordan, Amman 11733, Jordan; r.balasmeh@zuj.edu.jo; 9 AlSaidaly Scientific Bureau, Baghdad 10542, Iraq; abdulqader.fadhil10@icloud.com (A.F.A.); yasmeenib84@gmail.com (Y.I.); 10 Biology Department, College of Science, King Khalid University, Abha 61413, Saudi Arabia; aaldoaiss@kku.edu.sa (A.A.A.-D.); alshehri44@gmail.com (M.A.A.)

**Keywords:** diabetes, fasting blood glucose, intermittent fasting, Ramadan, dry fasting

## Abstract

Background: The level of fasting blood glucose (FBG) is influenced by several factors, including health status, genetics, and diet. Some studies have reported a beneficial effect of Ramadan Intermittent Fasting (RIF) on diabetic patients. However, clinical observations have shown that diabetes is exacerbated in some patients. Aim: This study aims to investigate the influence of RIF on the FBG level, a biomarker of hyperglycemia and diabetes, and to identify factors associated with variations in FBG levels during RIF among diabetic patients. Methods: This study is a cross-sectional study. We monitored the FBG levels of 181 type II diabetic patients over a two-month period, from 20 February to 20 April 2023, which represents the Islamic lunar months of Shaban (8th month) and Ramadan (9th month). Ramadan provides a prominent month of intermittent fasting practice for studying its physiological effects on diabetes. We collected clinical data from each participant, including demographic information, co-morbidities, and medications used during this period. Results: Based on our findings, diabetic patients were classified into three groups depending on the influence of RIF on FBG levels: the positively affected group (44%), whose average FBG levels were reduced; the neutrally affected group (24%), whose average FBG levels did not change; and the negatively affected group (32%), whose average FBG levels increased during the fasting month of Ramadan compared to the previous month. Furthermore, we found that the positive effect of RIF was more frequent among obese, non-geriatric, and male diabetic patients, while the negative effect of RIF was more frequent among patients who were not adhering to the medication. Conclusions: This study concludes that RIF affects FBG levels differently among diabetic patients. These findings should be taken into consideration when treating diabetic patients during the fasting month of Ramadan, and further studies are needed to identify (1) factors associated with inter-individual variation in the response to RIF and (2) those who are great candidates for RIF.

## 1. Introduction

Fasting is a practice that entails abstaining from food and/or drink for a designated duration, historically employed for religious, cultural, and health purposes. Intermittent fasting has gained popularity as a contemporary trend, embraced by individuals aiming to achieve weight loss, enhance their general well-being, and induce several anti-aging effects [1,2]. Ramadan fasting is a specific form of intermittent fasting where Muslim adults abstain from both food, drink, and medications (referred to as dry fasting [3]) from pre-dawn until sunrise throughout the month of Ramadan, setting it apart from general intermittent fasting practices [4].

Fasting blood glucose levels are a significant indicator of overall health. High levels can lead to a range of health problems, including the onset of type II diabetes [5]. Diabetes is a chronic disease characterized by elevated levels of blood sugar resulting from the inability of the body to produce or use insulin effectively. There are two main types of diabetes: type I and type II. Type I diabetes is an autoimmune disease that usually develops in childhood or adolescence and requires lifelong insulin therapy. Type II diabetes is the more common form of the disease and develops when the body becomes resistant to insulin or does not produce enough insulin to regulate blood sugar levels [6].

Research has shown that fasting has a positive effect on type II diabetes by decreasing blood glucose levels [7]. Studies have shown that intermittent fasting can improve insulin sensitivity and blood sugar control [8,9]. In addition, fasting has been shown to reduce inflammation, improve blood lipid profiles, and promote weight loss, which can help in the management of diabetes [10,11].

On the other hand, there are some reports that RIF can exacerbate diabetes and its complications [12,13,14]. However, the mechanism by which RIF affects diabetic patients is still not understood.

There are controversial results regarding the effect of RIF on diabetes. Therefore, this study aimed to investigate the effect of RIF on the level of FBG, one of the major biomarkers of diabetic control [15], and to identify factors associated with alterations of FBG levels during RIF.

## 2. Materials and Methods

### 2.1. Characteristics of the Participants 

Two hundred and thirty-two type II diabetic patients enrolled in this study, with patients attending various hospitals and clinics in different cities in the West Bank of Palestine (WBP), including Ramallah, Nablus, and Jenin, which are the largest cities in WBP. Diabetic patients were diagnosed by endocrinologists with a glycosylated hemoglobin (HbA1c) level > 6.5%, in accordance with the guidelines of the American Association of Diabetes [16], which are primarily followed in WBP.

The inclusion criteria for this study included individuals with type II diabetes who had intentions to fast during Ramadan 2023. The patients included in this study had independently made the decision to fast during the month of Ramadan, and our research did not influence their choice. Every participant patient provided informed consent to participate in this study. Prior to their participation, we informed them about the potential beneficial or negative effects of fasting, and they voluntarily decided to proceed with their fasting practices. Detailed information about each participant was checked to avoid the overlap of patients in this study among different hospitals and clinics. However, at the end of Ramadan, the data showed that 51 patients did not continue to fast the whole Ramadan month and did not provide information regarding the FBG and medication adherence (according to the data of 181 diabetic patients who were included in the analysis of this study).

The protocol of this study was approved by the ethical committee at An-Najah National University (Nablus, WBP) with an IRB number of Med. 4-2023/9.

### 2.2. Demographic Data

The demographic data and co-morbidities were collected using the medical records of the hospitals and clinics where the patients received medical care.

### 2.3. Attitude toward Ramadan Intermittent Fasting 

In this study, three questions were formulated to assess the participants’ attitudes towards Ramadan Intermittent Fasting (RIF) and intermittent fasting practices. The questions were administered to 30 diabetic patients to ensure clarity of meaning. The researchers collected the responses to these questions during direct meetings with the participating patients.

### 2.4. Fasting Blood Glucose Level

The levels of FBG for each patient were collected from the records of hospitals and clinics where the patient attended. A drop of blood was applied to a chemically treated, disposable ‘test-strip’, which was then put into an electronic blood glucose meter. The meter detected the reaction between the test strip and the blood and displayed the blood glucose level in mg/dL units. The average levels of FBG were calculated depending, at least, on 5 different readings of FBG levels during the month prior to fasting and 5 different readings during Ramadan month. The average FBG levels were collected from 29 February to 19 March 2023, representing the FBG level during the month prior to fasting. Additionally, the average FBG levels were calculated from 20 March to 20 April 2023, representing the FBG level during the fasting month.

### 2.5. Medication Adherence 

The medication adherence toward the antidiabetic drugs was assessed, as published previously [17,18], through a direct counseling session with the participants. In Ramadan, Muslims abstain from medications from sunrise to sunset. After the sunset, Muslims can start taking medications until the sunrise of the next day. 

### 2.6. Statistical Analysis 

Categorical data pertaining to various variables were represented using frequency (N) and percentages (%). To ascertain the statistical significance of differences in the proportions of “no benefits” and “negative effects”, which were coded as 0 = Yes and 1 = No, respectively, for independent variables, the Chi-Square test was employed. Subsequently, binary logistic regression was employed to assess the statistical significance of variations in the proportions of positive benefits derived from fasting during Ramadan, which were coded as 0 = Negative effects/No benefits and 1 = Positive benefits, in relation to independent variables. Thereafter, a multiple logistic regression analysis was conducted, incorporating independent variables that demonstrated significance in the binary logistic regression, in order to identify factors associated with positive benefits stemming from fasting during Ramadan. The significance level was set at a *p* value < 0.05. Statistical Package for the Social Sciences (SPSS) software (version 26, IBM, New York, NY, USA) was used in the statistical analysis of the study data.

**Figure 1:**
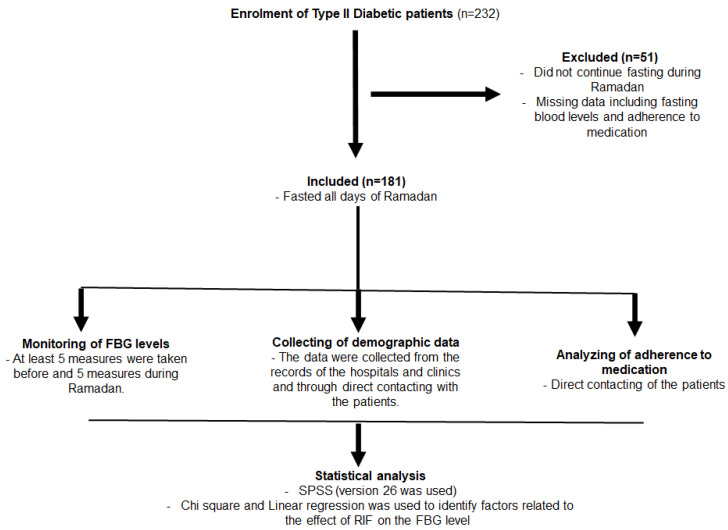
The flowchart of the study design.

The flowchart of the study design is illustrated in Figure 1.

## 3. Results

### 3.1. Demographic Data

Table 1 shows the demographic data of the participants: 44.2% were living in large cities, 55.6% were living in villages, and 2.2% were living in refugee camps. The majority of the participants were aged between 51 and 60 (28.7%), followed by those under 40 years old (24.9%), individuals aged between 61 and 70 (19.3%), those aged between 41 and 50 (18.2%), and a minority above 70 years old (8.8%). The distribution of participants based on sex was nearly equal, with 53% being males and 47% being females. A significant proportion of participants (40.3%) resided in families with three or fewer members. Regarding weight, 42% of participants were overweight, while 9.4% were obese. Conversely, 40.9% of participants had a normal weight, and only 7.7% were classified as underweight.

As shown in Table 1, more than half of participant patients (55.2%) have other medical conditions other than diabetes, including cardiovascular (35.4%), respiratory (6.1%), musculoskeletal (13.8%), digestive (7.2%), endocrine (5.5%), and psychological (6.1%) disorders.

### 3.2. Types of Medicines and Adherence before and during Ramadan

Table 2 shows the types of medications administered to diabetic patients during their fasting period. Most (55.8%) of the participants were on combined oral hypoglycemic agents without insulin therapy; among them, 22.7% of the patients were on metformin, sulphonylurea, and dipeptidyl peptidase IV inhibitors, while 32% of patients were on metformin alone.

### 3.3. FBG Values, Attitude, and Practice toward Ramadan Fasting among Diabetic Patients 

In this study, it was observed that 69.6% of the participating diabetic patients were able to fast throughout the entire Ramadan month in 2023, comprising 29 days, whereas the remaining patients (30.4%) were unable to continue fasting (Table 3). In addition, 43.6% of the participant patients expressed that Ramadan fasting is challenging for diabetic patients (Table 3).

**Figure 2:**
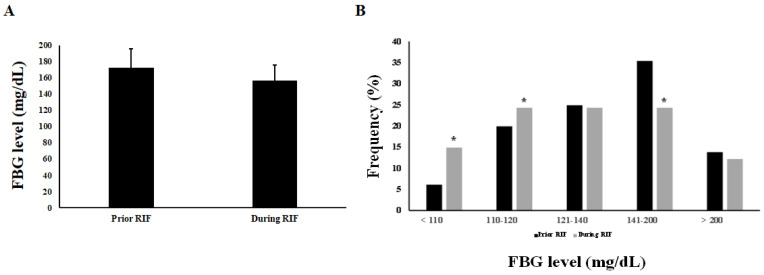
Comparison of the average and frequency levels of FBG prior to and during RIF. Part (**A**) represents the total FBG average ± SD prior to and during RIF. Part (**B**) represents the comparison of the frequency of FBG interval ranges among diabetic patients before and during RIF. Data are represented as frequencies (%) of diabetic patients within the category of FBG level. “*” indicates statistical significance (*p* < 0.05, Chi-square).

Figure 2 compares FBG levels before and during fasting. The total average ± SD of FBG in diabetic patients was 172.18 ± 23.62 ng/dL prior to RIF and 156.24 ± 19.12 ng/dL during RIF (Figure 2A). We found in this study that the percentage of diabetic patients with an average FBG level of less than 110 ng/dL is significantly higher (14.9%) during Ramadan fasting compared to only 6.1% of participants who had an average FBG level of less than 110 ng/dL before the Ramadan month (Figure 2B). Additionally, the frequency percentage of participants with an average FBG value between 110 and 120 ng/dL is significantly higher (24.3%, *p* < 0.05) during Ramadan fasting compared to 19.9% of participants with an average FBG value between 110 and 120 ng/dL before Ramadan (Figure 2B).

On the other hand, the frequency percentage of FBG values indicating uncontrolled diabetes significantly decreased (*p* < 0.05) for the diabetic patients during the fasting month of Ramadan. During this period, 24.3% and 12.2% of participants had average FBG levels ranging from 140 to 200 and >200 ng/dL, respectively. In comparison, before the fasting month of Ramadan, 35.4% and 13.8% had average FBG levels in the range of 140–200 and >200 ng/dL, respectively (Figure 2B).

### 3.4. Effects of Fasting for Ramadan on FBG Value

**Figure 3:**
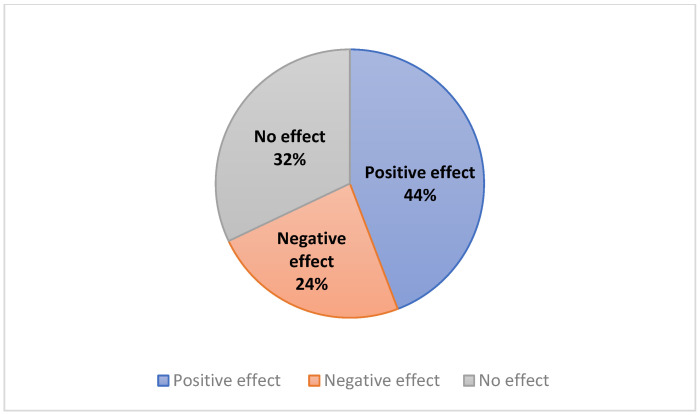
Effect of Ramadan fasting on the level of FBG among diabetic patients. The effect of Ramadan fasting on the level of FBG was assessed by comparing the average FBG level during and before Ramadan. When the level of FBG was increased during Ramadan, it was considered a positive effect, while when the level of FBG was decreased during Ramadan, it was considered a negative effect. “No effect” means that the average levels of FBG did not change before and during Ramadan month more than 20 ng/dL.

Results showed that RIF affects FBG values differently among the participating diabetic patients, as follows: 44% of diabetic patients demonstrated a decrease (positive effect), while 32% showed an increase (negative effect) in their FBG levels during Ramadan (Figure 3). Furthermore, 24% of the participant patients exhibited FBG values that were almost comparable (not changed more than 20 ng/dL) to those observed one month before Ramadan (Figure 3).

### 3.5. Factors Affecting FBG Levels during the Fasting of Ramadan 

Table 4 presents an overview of the distribution and relationship between the absence of health benefits (i.e., no benefits) and the presence of adverse effects associated with fasting during Ramadan (i.e., negative effects) among participants with varying socio-demographic and clinical characteristics. Subsequent analysis has revealed a significant relationship between sex and CVDs with regards to experiencing negative effects during fasting in Ramadan (i.e., negative effects) (*p* < 0.05). Specifically, it was observed that female participants accounted for 69.8% of cases, and those without CVDs constituted 79.1% of instances, both demonstrating significantly higher proportions of negative effects arising from fasting during Ramadan. Moreover, a significant association between medication adherence during Ramadan and the occurrence of no benefits or negative effects was identified (*p* < 0.05). Notably, participants who were non-adherent to their prescribed medications during Ramadan significantly exhibited the highest percentages of experiencing no benefits (87.2%) and negative effects from fasting during Ramadan (100%).

Table 5 shows the univariate analysis of socio-demographic and clinical characteristics associated with the likelihood of experiencing the positive health benefits of fasting during Ramadan. Subsequently, age, sex, positive attitude toward Ramadan Intermittent Fasting (RIF), CVDs, psychological diseases, and receiving antihypertensive agents were significantly (*p* < 0.05) associated with decreased odds of positive benefits of fasting during Ramadan. Participants aged over 50 years old were found to be significantly less likely to derive positive health benefits from fasting during Ramadan. Specifically, participants in the age groups of 51–60 years old (Odds Ratio [OR] = 0.224, 95% Confidence Interval [CI] [0.066–0.762]), 61–70 years old (OR = 0.133, 95% CI [0.032–0.556]), and those over 70 years old (OR = 0.082, 95% CI [0.012–0.573]) exhibited diminished odds of experiencing positive health benefits. Furthermore, female participants and those with a negative attitude toward RIF were also significantly less likely to gain positive health benefits (OR = 0.383, 95% CI [0.163–0.900] and OR = 0.001, 95% CI [0.000–0.187], respectively). 

Additionally, individuals who reported not having cardiovascular diseases (CVDs) or psychological disorders were significantly less likely to derive positive health benefits (OR = 0.221, 95% CI [0.058–0.847] and OR = 0.166, 95% CI [0.029–0.955], respectively). In contrast, participants not receiving antihypertensive agents were significantly less likely to experience positive benefits compared to those who received such medication (OR = 0.058, 95% CI [0.007–0.476]). Conversely, weight status showed a significant (*p* < 0.05) association with increased odds (likelihood) of deriving positive health benefits during Ramadan. Specifically, individuals categorized as normal weight, overweight, and obese were significantly more likely to experience positive health benefits (OR = 18.020, 95% CI [2.309–140.621]; OR = 8.778, 95% CI [1.117–68.958]; OR = 20.932, 95% CI [1.959–223.663], respectively) (Table 5).

In the context of multivariate analysis, after adjusting for covariates as presented in Table 6, several factors were found to be significantly associated with the positive health benefits derived from fasting during Ramadan. Notably, sex, the presence of CVDs, and the receipt of antihypertensive agents emerged as significant predictors of these benefits (*p* < 0.05). Female participants and individuals without CVDs were observed to be significantly less likely to experience positive health benefits from fasting during Ramadan. Specifically, female participants exhibited reduced odds of gaining positive health benefits (Odds Ratio [OR] = 0.469, 95% Confidence Interval [CI] [0.235–0.935]). Furthermore, a significant association (*p* < 0.05) was established between the receipt of antihypertensive agents and the likelihood of experiencing positive health benefits. Participants not receiving antihypertensive agents exhibited decreased odds of attaining positive health benefits compared to their counterparts who were prescribed such medication (OR = 0.228, 95% CI [0.062–0.837]).

However, it is noteworthy that age and weight, while not reaching statistical significance, displayed certain trends in their association with positive health benefits during multivariate analysis. Specifically, participants within the age groups of 61–70 years old (Odds Ratio [OR] = 0.195, 95% Confidence Interval [CI] [0.063–0.608], *p* < 0.05) and those aged over 70 years (OR = 0.215, 95% CI [0.050–0.930], *p* < 0.05) exhibited a significant decrease in the odds of experiencing positive health benefits, despite the lack of overall statistical significance. Conversely, individuals classified as normal weight and obese displayed a significantly heightened likelihood of experiencing positive health benefits (OR = 5.683, 95% CI [1.214–26.609], *p* < 0.05; OR = 6.403, 95% CI [1.033–39.700], *p* < 0.05, respectively), as indicated in Table 6.

## 4. Discussion

A significant number of Muslims with diabetes choose to observe fasting during Ramadan. Moreover, there have been previous reports suggesting an overall positive effect of RIF among diabetic patients [7,8,9]. However, endocrinologists have observed that some patients experience an exacerbation of diabetes symptoms, characterized by elevated FBG levels and fatigue, during the fasting month of Ramadan [12,13,14]. Therefore, these observations highlight the need for a comprehensive understanding of the diverse effects of RIF on diabetic patients.

In this study, we closely monitored FBG levels among diabetic patients who were fasting before and during Ramadan. Our findings indicate that RIF affects FBG level changes among fasting diabetic patients during Ramadan in different ways. The effects of RIF on diabetic patients can be categorized into three groups: (1) a positive effect, where FBG levels further decrease during fasting; (2) a negative effect, where FBG levels further increase during fasting; and (3) a neutral or no effect, where FBG levels remain unchanged during fasting compared to the previous month when patients did not fast.

This study represents the first report on the variation in the effect of RIF on FBG levels among diabetic patients. These results have important implications for physicians and endocrinologists in the treatment of diabetes during Ramadan, highlighting the need to consider individual patient responses to RIF. Treatment plans and fasting recommendations during Ramadan should be tailored accordingly. Further studies are still necessary to investigate and identify the factors contributing to the elevation of FBG levels among diabetic patients during RIF. 

In this study, we monitored the level of FBG among participant patients for two continuous months: one month prior to fasting and one month during the fasting period. We used FBG level rather than HbA1c as an indicator of the effect of RIF on diabetic patients since the value of HbA1c represents the level of blood glucose during the last three months [19]. Therefore, the effect of RIF, which is for one month, on the value of HbA1c can be masked by the other two months when patients generally do not fast. 

Diabetic patients who got a positive effect of RIF formed the group with the highest percentage (44%) in comparison with the other two groups. Additionally, the analysis of the FBG levels of the diabetic participants in this study showed that the frequency of controlled diabetes with FBG levels <120 ng/dL was increased, while the frequency of uncontrolled diabetes with FBG levels >120 ng/dL was decreased among fasting diabetic patients. The available data suggests that a considerable percentage of diabetic patients experience beneficial effects from RIF. 

Recent research indicates that there is growing support for the potential benefits of intermittent fasting, including the reduction of body weight and the improvement of insulin sensitivity [8]. Observers of RIF usually reduce their food intake, which can lead to decreased FBG levels [20]. These findings are in agreement with previous studies that showed that RIF has beneficial effects on diabetic patients [21,22,23]. However, in this study, we investigated factors, including the collected clinical data and patients’ attitudes and practices, that are associated with the positive beneficial effects of RIF, and we found in this study that aging reduced the positive effects of RIF on FBG among diabetic patients. This can be explained by the fact that there are physiological changes that occur during aging, such as a decrease in metabolism [24], that interact with RIF and influence the level of FBG. Additionally, we found in this study that obese diabetic patients got more positive effects from RIF than non-obese patients. It was reported that RIF reduces body weight [25], which hence decreases insulin resistance and reduces blood glucose levels [8].

**Figure 4:**
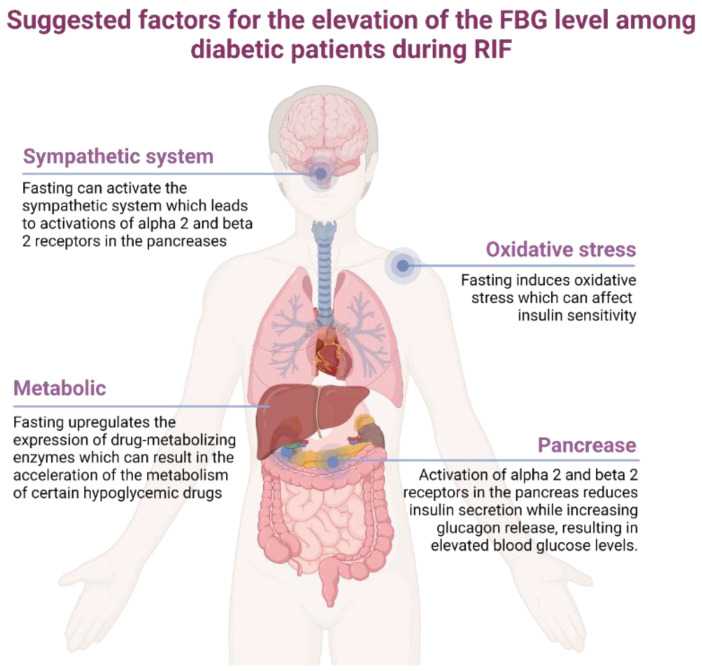
Suggested factors for the elevation of FBG levels among diabetic patients during RIF.

Moreover, we found that not all diabetic patients gained the beneficial effect of RIF; some diabetic patients (24% of the total participants in this study) had elevated levels of FBG, which can worsen the symptoms and complications of diabetes. The following are some suggested factors that can explain the elevation of the FBG level among diabetic patients (Figure 4): (1) Fasting upregulates the expression of drug-metabolizing enzymes, which can result in the acceleration of the metabolism of certain hypoglycemic drugs and hence reduce the response of these drugs’ effect [26]; (2) Fasting can activate the sympathetic system, which leads to activations of alpha 2 and beta 2 receptors in the pancreas and hence reduces insulin while increasing glucagon release and elevating blood glucose levels [27]. Furthermore, (3) RIF induces oxidative stress, which can affect insulin sensitivity [28]. Additionally, it was reported that the frequency of medication administration is altered during Ramadan, which may affect the drug response among diabetic patients [29].

In this study, our objective was to identify potential clinical factors associated with the negative impact of RIF on FBG levels. It is found in the current study that the negative effect of RIF was more frequent among female than male diabetic patients (*p* < 0.05). On the other hand, the positive effect of RIF was more frequent among male than female diabetic patients. The exact explanation of this sex-dependent variation in the effect of RIF on FBG level is unclear to us. It might be due to the differences in the effect of sexual hormones on blood glucose levels [30] or to other reasons that need further investigation. Furthermore, the results of this study indicate the importance of medication adherence in the control of diabetes and the prevention of its exacerbation [31,32] by preventing the negative effect of RIF on the level of FBG.

## 5. Conclusions

In this study, our main focus centered on the monitoring of fasting blood glucose levels among diabetic patients throughout Ramadan. The results uncovered significant inter-individual variability in how diabetic patients respond to RIF. These findings of this study represent a step forward in comprehending the effects of intermittent fasting, subsequently offering valuable guidance for the optimization of therapy among diabetic patients, specifically during the month of Ramadan.

## Figures and Tables

**Table 1:** Demographic and clinical characteristics of study participants.

Demographic or Clinical	Frequency n (%)
Residency Location	
Large City	80 (44.2)
Village	97 (55.6)
Palestinian Refugee Camp	4 (2.2)
Age	
<40 Years old	45 (24.9)
40–50 Years old	33 (18.2)
51–60 Years old	52 (28.7)
61–70 Years old	35 (19.3)
>70 Years old	16 (8.8)
Sex	
Male	96 (53.0)
Female	85 (47.0)
Number of Family Members	
Living alone	8 (4.4)
1–3 Family Members	73 (40.3)
4–5 Family Members	54 (29.8)
>5 Family Members	46 (25.4)
Weight (depending on the BMI value)	
Under Weight	14 (7.7)
Normal Weight	74 (40.9)
Overweight	76 (42.0)
Obese	17 (9.4)
Other Diseases?	
Yes	100 (55.2)
No	81 (44.8)
Respiratory Diseases?	
Yes	11 (6.1)
No	170 (93.9)
Cardiovascular Diseases?	
Yes	64 (35.4)
No	117 (64.6)
Musculoskeletal Diseases?	
Yes	25 (13.8)
No	156 (86.2)
Digestive Diseases?	
Yes	13 (7.2)
No	168 (92.8)
Endocrine Diseases?	
Yes	10 (5.5)
No	171 (94.5)
Psychological Diseases?	
Yes	11 (6.1)
No	170 (93.9)

**Table 2:** Medication utilization pattern profile.

Medications	Frequency n (%)
Antibiotics	
Yes	3 (1.7)
No	178 (98.3)
Aspirin	
Yes	14 (7.7)
No	167 (92.3)
Anti-hyperlipidemic	
Yes	21 (11.6)
No	160 (88.4)
Anti-hypertensive	
Yes	15 (8.3)
No	166 (91.7)
Anti-Diabetic Treatment Modalities	
None	8 (4.4)
Oral Hypoglycemic Drugs (OHDs)	101 (55.8)
Combination of OHDs and Insulin	18 (9.9)
Insulin	54 (29.8)
Anti-Diabetic Treatment Regimen	
None	9 (5.0)
Metformin	58 (32.0)
Metformin + SUs and/or DPP4 Inhibitors	41 (22.7)
Insulin	54 (29.8)
Insulin + Metformin	11 (6.1)
Insulin + Metformin and/or DPP4 Inhibitors	8 (4.4)
Medication Adherence before Ramadan	
Yes	169 (93.4)
No	12 (6.6)
Medication Adherence during Ramadan	
Yes	169 (93.4)
No	12 (6.6)

**Table 3:** Fasting during Ramadan.

Variable	Frequency n (%)
Did you fast the whole Ramadan month?	
Yes	126 (69.6)
No	55 (30.4)
Is fasting easy during Ramadan?	
Yes	102 (56.4)
No	79 (43.6)
Do you think that RIF has a negative effect on diabetic patients?	
Yes	73 (40.3)
No	108 (59.7)

**Table 4:** Distribution of unchanged FBG and negative effects of fasting during Ramadan among socio-demographic and clinical characteristics of participants (N = 101).

Variables: (Socio-Demographic and Clinical Characteristics)	Unchanged FBG Level (Yes) N = 58	*p* Value	Negative Effects (Yes) N = 43	*p* Value
Residency Location		0.165		0.364
Large Cities	26 (44.8)		17 (39.5)	
Small Cities	29 (50.0)		26 (60.5)	
Palestinian Refugee Camp	3 (5.2)		0 (0.0)	
Age		0.170		0.840
<40 Years old	10 (17.2)		10 (23.3)	
40–50 Years old	13 (22.4)		6 (14.0)	
51–60 Years old	15 (25.9)		14 (32.6)	
61–70 Years old	16 (27.6)		8 (18.6)	
>60 Years old	4 (6.9)		5 (11.6)	
Sex		0.475		**0.001**
Male	33 (56.9)		13 (30.2)	
Female	25 (43.1)		30 (69.8)	
Number of Family Members		0.324		0.699
Living alone	3 (5.2)		3 (7.0)	
1–3 Family members	27 (46.6)		17 (39.5)	
4–5 family members	12 (20.7)		14 (32.6)	
>5 family members	16 (27.6)		9 (20.9)	
Weight (Depending on BMI)		0.093		0.774
Under weight	8 (13.8)		3 (7.0)	
Normal weight	19 (32.8)		15 (34.9)	
Over weight	27 (46.6)		20 (46.5)	
Obese	4 (6.9)		5 (11.6)	
Other Diseases?		0.989		0.095
Yes	32 (55.2)		19 (44.2)	
No	26 (44.8)		57 (41.3)	
Respiratory Diseases?		0.325		0.654
Yes	5 (8.6)		2 (4.7)	
No	53 (91.4)		41 (95.3)	
Cardiovascular Diseases?		0.866		**0.023**
Yes	20 (34.5)		9 (20.9)	
No	38 (65.5)		34 (79.1)	
Musculoskeletal Diseases?		0.641		0.975
Yes	7 (12.1)		6 (14.0)	
No	51 (87.9)		37 (86)	
Digestive Diseases?		0.051		0.196
Yes	1 (1.7)		5 (11.6)	
No	57 (98.3)		38 (88.4)	
Endocrine Diseases		0.579		0.774
Yes	4 (6.9)		2 (4.7)	
No	54 (93.1)		41 (95.3)	
Psychological Diseases		0.726		0.238
Yes	3 (5.2)		1 (2.3)	
No	55 (94.8)		42 (97.7)	
Aspirin		0.376		0.080
Yes	3 (5.2)		6 (14.0)	
No	55 (94.8)		37 (86.0)	
Anti-Hyperlipidemic		0.717		0.101
Yes	6 (10.3)		8 (18.6)	
No	52 (89.7)		35 (81.4)	
Anti-Hypertensive		0.105		0.721
Yes	2 (3.4)		3 (7.0)	
No	56 (96.6)		40 (93.0)	
Anti-Diabetic Treatment Modalities		0.740		0.365
None	3 (5.2)		1 (2.3)	
OHDs	29 (50.0)		24 (55.8)	
Combines OHDs and Insulin	7 (12.1)		7 (16.3)	
Insulin alone	19 (32.8)		11 (25.6)	
Anti-Diabetic Treatment Regimen		0.760		0.078
None	3 (5.2)		1 (2.3)	
Metformin	17 (29.3)		9 (20.9)	
Metformin + SUs and/or DPP4Is	13 (22.4)		15 (34.9)	
Insulin	19 (32.8)		11 (25.6)	
Insulin + Metformin	2 (3.4)		5 (11.6)	
Insulin + Metformin and/or DPP4Is	4 (6.9)		2 (4.7)	
Medication Adherence before Ramadan		0.167		0.194
Yes	6 (10.3)		1 (2.3)	
No	52 (89.7)		42 (97.7)	
Medication Adherence during Ramadan		0.087		**0.035**
Yes	7 (12.1)		0 (0.0)	
No	51 (87.9)		43 (100.0)	

*p* values obtained from the Chi-Square Test. Bold *p* values are statistically significant (chi-square test). BMI: body mass index. OHDs: oral hypoglycemic drugs. SUs: sulfonylureas. DDP4Is: dipeptidyl peptidase 4 inhibitors.

**Table 5:** Univariate analysis of factors associated with the positive benefits of fasting during Ramadan (N = 181).

Variable	Odds Ratio with 95% CI	*p* Value
Residency Location		0.29
Large Cities	Reference	
Small Cities	1.006 (0.462–2.192)	0.998
Palestinian Refugee Camp	0.068 (0.002–1.994)	0.119
Age		**0.043**
<40 Years old	Reference	
40–50 Years old	0.358 (0.106–1.216)	0.1
51–60 Years old	0.224 (0.066–0.762)	**0.017**
61–70 Years old	0.133 (0.032–0.556)	**0.006**
>70 Years old	0.082 (0.012–0.573)	**0.012**
Weight		**0.023**
Underweight	Reference	
Normal Weight	18.020 (2.309–140.621)	**0.006**
Overweight	8.778 (1.117–68.958)	**0.039**
Obese	20.932 (1.959–223.663)	**0.012**
Sex		**0.028**
Male	Reference	
Female	0.383 (0.163–0.900)	
Number of Family Members		0.573
Living alone	Reference	
1–3 Family Members	2.962 (0.320–27.405)	0.339
4–5 Family Members	4.655 (0.463–46.764)	0.191
>5 Family Members	3.740 (0.367–38.136)	0.266
Fasting the whole Ramadan?		
Yes	0.583 (0.177–1.918)	0.375
No	Reference	
Positive attitude toward RIF		
Yes	Reference	**0.009**
No	0.001 (0.000–0.187)	
Other Diseases?		
Yes	Reference	0.315
No	2.152 (0.482–9.599)	
Cardiovascular Diseases?		
Yes	Reference	0.088
No	0.221 (0.058–0.847)	
Respiratory Diseases?		
Yes	Reference	0.178
No	0.236 (0.029–1.926)	
Musculoskeletal Diseases?		
Yes	Reference	0.331
No	0.550 (0.165–1.835)	
Digestive Diseases?		
Yes	Reference	0.224
No	0.369 (0.074–1.840)	
Endocrine Diseases?		0.802
Yes	Reference	
No	0.800 (0.139–4.598)	
Psychological Diseases?		
Yes	Reference	0.054
No	0.166 (0.029–0.955)	
Aspirin?		
Yes	Reference	0.396
No	2.913 (0.247–34.346)	
Anti-Hyperlipidemic?		
Yes	Reference	0.734
No	1.299 (0.287–5.889)	
Antihypertensive?		
Yes	Reference	**0.078**
No	0.058 (0.007–0.476)	
Anti-Diabetic Treatment Modalities:		0.998
None	Reference	
Oral Hypoglycemic Drugs (OHDs)	216.3 (0.000)	1
Combination of OHDs and Insulin	2.922 (0.000)	1
Insulin alone	1.204 (0.196–7.406)	0.842
Anti-Diabetic Treatment Regimen:		0.224
None	Reference	
Metformin	0.000 (0.000)	1
Metformin + SUs and/or DPP4 Inhibitors	0.000 (0.000)	1
Insulin	0.000 (0.000)	1
Insulin + Metformin	0.748 (0.000)	1
Insulin + Metformin and/or DPP4 Inhibitors	0.103 (0.000)	1
Medication Adherence before Ramadan		
Yes	1.503 (0.182–12.421)	0.705
No	Reference	
Medication Adherence during Ramadan		
Yes	2.970 (0.330–26.772)	
No	Reference	0.332

*p* values obtained from binary logistic regression. Bold *p* values are statistically significant (binary logistic regression).

**Table 6:** Multivariate analysis of factors associated with the positive health benefits of fasting during Ramadan.

Variable	Odds Ratio with 95% CI	*p* Value
Age		0.067
<40 Years old	Reference	
40–50 Years old	0.491 (0.181–1.328)	0.161
51–60 Years old	0.407 (0.160–1.038)	0.060
61–70 Years old	0.195 (0.063–0.608)	**0.005**
>70 Years old	0.215 (0.050–0.930)	**0.040**
Weight		**0.087**
Underweight	Reference	
Normal Weight	5.683 (1.214–26.609)	**0.027**
Overweight	3.263 (0.700–15.204)	0.132
Obese	6.403 (1.033–39.700)	**0.046**
Sex		**0.031**
Male	Reference	
Female	0.469 (0.235–0.935)	
Positive attitude toward RIF		
Yes	Reference	
No	0.732 (0.362–1.480)	0.385
Cardiovascular Diseases?		
Yes	Reference	
No	0.472 (0.274–0.896)	0.071
Psychological Diseases?		
Yes	Reference	
No	0.283 (0.066–1.219)	0.090
Antihypertensive?		
Yes	Reference	
No	0.328 (0.062–0.837)	0.056

*p* values obtained from multiple logistic regression. Bold *p* values are statistically significant (multiple logistic regression).

## Data Availability

Data are available with the corresponding author upon request.

## References

[1] Visioli F., Mucignat-Caretta C., Anile F., Panaite S.A. (2022). Traditional and Medical Applications of Fasting. Nutrients.

[2] de Cabo R., Carmona-Gutierrez D., Bernier M., Hall M.N., Madeo F. (2014). The search for antiaging interventions: From elixirs to fasting regimens. Cell.

[3] Papagiannopoulos-Vatopaidinos I.E., Papagiannopoulou M., Sideris V. (2020). Dry Fasting Physiology: Responses to Hypovolemia and Hypertonicity. Complement. Med. Res..

[4] Madkour M., Giddey A.D., Soares N.C., Semreen M.H., Bustanji Y., Zeb F., Halwani R., Faris M.E. (2022). Ramadan diurnal intermittent fasting is associated with significant plasma metabolomics changes in subjects with overweight and obesity: A prospective cohort study. Front. Nutr..

[5] Park S.K., Jung J.Y., Oh C.M., Choi J.M., Kim M.H., Ha E., Ryoo J.H. (2021). Fasting glucose level and the risk of incident osteoporosis in the Koreans. Bone.

[6] Silva J.A.D., Souza E.C.F., Echazu Boschemeier A.G., Costa C., Bezerra H.S., Feitosa E. (2018). Diagnosis of diabetes mellitus and living with a chronic condition: Participatory study. BMC Public Health.

[7] Saeed M., Ali M., Zehra T., Haider Zaidi S.A., Tariq R. (2021). Intermittent Fasting: A User-Friendly Method for Type 2 Diabetes Mellitus. Cureus.

[8] Yuan X., Wang J., Yang S., Gao M., Cao L., Li X., Hong D., Tian S., Sun C. (2022). Effect of Intermittent Fasting Diet on Glucose and Lipid Metabolism and Insulin Resistance in Patients with Impaired Glucose and Lipid Metabolism: A Systematic Review and Meta-Analysis. Int. J. Endocrinol..

[9] Vasim I., Majeed C.N., DeBoer M.D. (2022). Intermittent Fasting and Metabolic Health. Nutrients.

[10] Aly S.M. (2014). Role of intermittent fasting on improving health and reducing diseases. Int. J. Health Sci..

[11] Shehab A., Abdulle A., El Issa A., Al Suwaidi J., Nagelkerke N. (2012). Favorable changes in lipid profile: The effects of fasting after Ramadan. PLoS ONE.

[12] Salti I., Benard E., Detournay B., Bianchi-Biscay M., Le Brigand C., Voinet C., Jabbar A. (2004). A population-based study of diabetes and its characteristics during the fasting month of Ramadan in 13 countries: Results of the epidemiology of diabetes and Ramadan 1422/2001 (EPIDIAR) study. Diabetes Care.

[13] Al-Arouj M., Assaad-Khalil S., Buse J., Fahdil I., Fahmy M., Hafez S., Hassanein M., Ibrahim M.A., Kendall D., Kishawi S. (2010). Recommendations for management of diabetes during Ramadan: Update 2010. Diabetes Care.

[14] Ibrahim M., Abu Al Magd M., Annabi F.A., Assaad-Khalil S., Ba-Essa E.M., Fahdil I., Karadeniz S., Meriden T., Misha’l A.A., Pozzilli P. (2015). Recommendations for management of diabetes during Ramadan: Update 2015. BMJ Open Diabetes Res. Care.

[15] Long J., Yang Z., Wang L., Han Y., Peng C., Yan C., Yan D. (2020). Metabolite biomarkers of type 2 diabetes mellitus and pre-diabetes: A systematic review and meta-analysis. BMC Endocr. Disord..

[16] American Diabetes A. (2021). Standards of Medical Care in Diabetes-2021 Abridged for Primary Care Providers. Clin. Diabetes.

[17] Alqarni A.M., Alrahbeni T., Qarni A.A., Qarni H.M.A. (2019). Adherence to diabetes medication among diabetic patients in the Bisha governorate of Saudi Arabia—A cross-sectional survey. Patient Prefer. Adherence.

[18] Ashur S.T., Shamsuddin K., Shah S.A., Bosseri S., Morisky D.E. (2015). Reliability and known-group validity of the Arabic version of the 8-item Morisky Medication Adherence Scale among type 2 diabetes mellitus patients. East Mediterr. Health J..

[19] Sherwani S.I., Khan H.A., Ekhzaimy A., Masood A., Sakharkar M.K. (2016). Significance of HbA1c Test in Diagnosis and Prognosis of Diabetic Patients. Biomark. Insights.

[20] Harbuwono D.S., Sazli B.I., Kurniawan F., Darmowidjojo B., Koesnoe S., Tahapary D.L. (2021). The impact of Ramadan fasting on Fetuin-A level in type 2 diabetes mellitus. Heliyon.

[21] Assy M.H., Awd M., Elshabrawy A.M., Gharieb M. (2019). Effect of Ramadan fasting on incidence of cerebrovascular stroke in Egyptian patients with Type 2 Diabetes Mellitus. Diabetes Res. Clin. Pract..

[22] Bener A., Yousafzai M.T. (2014). Effect of Ramadan fasting on diabetes mellitus: A population-based study in Qatar. J. Egypt Public Health Assoc..

[23] Gad H., Al-Nassr N., Mohammed I., Khan A., MacDonald R., Mussleman P., Malik R.A. (2022). Effect of Ramadan fasting in patients with type 2 diabetes mellitus treated with sodium-glucose cotransporter 2 inhibitors: A systematic review and meta-analysis. J. Diabetes Investig..

[24] Roberts S., Rosenberg I. (2006). Nutrition and aging: Changes in the regulation of energy metabolism with aging. Physiol. Rev..

[25] Correia J.M., Santos I., Pezarat-Correia P., Silva A.M., Mendonca G.V. (2021). Effects of Ramadan and Non-ramadan Intermittent Fasting on Body Composition: A Systematic Review and Meta-Analysis. Front. Nutr..

[26] Balasmeh R., Jarrar Y., Al-Sheikh I., Alshaiah H., Jarrar Q., Alani R., Abudahab S. (2022). Effects of Fasting and Phoenix dactylifera on the Expression of Major Drug- Metabolizing Enzymes in the Mouse Livers. Curr. Drug Metab..

[27] Wang M., Wang Q., Whim M.D. (2016). Fasting induces a form of autonomic synaptic plasticity that prevents hypoglycemia. Proc. Natl. Acad. Sci. USA.

[28] Faris M.A., Hussein R.N., Al-Kurd R.A., Al-Fararjeh M.A., Bustanji Y.K., Mohammad M.K. (2012). Impact of ramadan intermittent fasting on oxidative stress measured by urinary 15-f(2t)-isoprostane. J. Nutr. Metab..

[29] Jarrar Y. (2011). Pharmaceutical practice and selling of drugs during Ramadan. Libyan J. Med..

[30] Brockman N.K., Sigal R.J., Kenny G.P., Riddell M.C., Perkins B.A., Yardley J.E. (2020). Sex-Related Differences in Blood Glucose Responses to Resistance Exercise in Adults with Type 1 Diabetes: A Secondary Data Analysis. Can. J. Diabetes.

[31] Garcia-Perez L.E., Alvarez M., Dilla T., Gil-Guillen V., Orozco-Beltran D. (2013). Adherence to therapies in patients with type 2 diabetes. Diabetes Ther..

[32] Wabe N.T., Angamo M.T., Hussein S. (2011). Medication adherence in diabetes mellitus and self management practices among type-2 diabetics in Ethiopia. N. Am. J. Med. Sci..

